# Immunometabolism unveiled: Pioneering breakthroughs in cancer therapeutics

**DOI:** 10.1002/1878-0261.13670

**Published:** 2024-06-13

**Authors:** Eric Eldering, Jean‐Ehrland Ricci

**Affiliations:** ^1^ Department of Experimental Immunology Amsterdam UMC location University of Amsterdam Amsterdam The Netherlands; ^2^ Cancer Immunology Amsterdam Institute for Infection and Immunity Amsterdam The Netherlands; ^3^ Cancer Immunology Cancer Center Amsterdam Amsterdam The Netherlands; ^4^ Université Côte d'Azur, INSERM, C3M Nice France; ^5^ Equipe labellisée Ligue Contre le Cancer Nice France

## Abstract

The field of immunometabolism cannot be considered ‘emerging’ anymore; it is at the moment one of the most active and rapidly evolving areas of biomedical research. Its hottest zone is cancer immunometabolism. This is partly due to the clinical application of immunotherapy, with either antibodies (checkpoint blockade) or cellular therapies (e.g., CAR‐T cells). In addition, the proliferating tumor cells create a nutrient‐deprived microenvironment that impairs the metabolic fitness and functionality of infiltrating immune cells such as T cells, NK cells, and macrophages. The key concepts are bidirectional metabolic signaling, plus the conviction that a better understanding of these processes will improve current immunotherapies, and foster new tools and targets for treatment. This collection of reviews will address various exciting aspects from junior and established scientists in the field.

## Graphical abstract

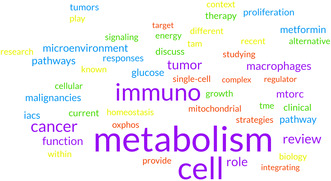

In the pursuit of conquering cancer, the quest for novel cures has never been more imperative. Recent breakthroughs have unveiled a striking revelation: Cancer cells exhibit peculiar ways of consuming and producing energy, challenging the conventional understanding of cellular metabolism. As we delve into this metabolic enigma, profound realization surfaces—not only do cancer cells undergo metabolic rewiring, but also the foot soldiers of our immune system—must also recalibrate their metabolic machinery to effectively combat their adversary.

The conventional view of cellular energy metabolism painted a simplistic portrait, yet the discovery that cancer cells harbor distinct metabolic signatures has ignited a paradigm shift. Unlike their nonmalignant counterparts, cancer cells often favor anaerobic glycolysis, commonly known as the ‘Warburg effect’, to fuel their rapid proliferation. The Warburg effect is defined by the increased uptake of glucose plus incomplete oxidation or fermentation into lactate in the presence of oxygen [1], which was thought to be the consequence of defective mitochondria. It has now become clear that this metabolic alteration represents only the tip of the iceberg with regard to the metabolic rearrangements that accompany malignant transformation, which involve not only aerobic glycolysis but also an increased requirement of amino acids, lipid synthesis, and NAD^+^/NADH homeostasis. [2]. In addition, and opposed to what Warburg et al. [3] suggested, it was established that despite engaging in aerobic glycolysis, cancer cells consume oxygen at levels comparable with normal tissue. Indeed, a recent article showed that in murine models, cancer cells predominantly utilize glutamine to fuel the tricarboxylic acid cycle (TCA) cycle while the interspersed immune cells mostly import glucose [4]. Respiration is also needed for tumor initiation, as tumor cells with impaired oxidative phosphorylation (OxPhos) due to the depletion of mitochondrial DNA (mtDNA) exhibit increased tumor latency upon subcutaneous transplantation. In fact, cells derived from these tumors acquire host mtDNA to regain the ability to do respiration, providing compelling evidence that respiration is required and selected for in tumorigenesis [5].

Despite the gradual retreat of the Warburg effect as the prime metabolic alteration in cancer, the critical question remains: Can we exploit these metabolic peculiarities as vulnerabilities to develop novel cancer therapies, and/or complement existing treatments?

Equally captivating is the realization that the immune system, our body's sentinel against malignancy, also undergoes metabolic modulation similar to that orchestrated by cancer. For immune cells to function optimally, they too must reprogram their metabolic pathways. The delicate balance between effector and regulatory functions hinges on the metabolic fitness of immune cells, thereby influencing the success or failure of immunotherapies. A beacon of hope and innovation is the emerging field of immunometabolism in cancer research. This developed almost in parallel with the clinical success of cancer immunotherapy, and also from the realization that its frequent failures were linked with the intricate metabolic interplay between cancer and immune cells. Appreciating the profound impact of metabolic reprogramming in both cancer and immune cells unveils a myriad of opportunities for therapeutic intervention. It invites us to explore not only how cancer cells exploit unique metabolic pathways but also how we can strategically manipulate the metabolic dynamics of immune cells to enhance their antitumor capabilities.

In this collection of reviews, we have not attempted to cover all aspects of the emerging but already vast field of immunometabolism. We have focused on some areas that were not yet extensively covered by the many excellent reviews that appeared in recent years, mixing contributions from junior and established scientists in the field (Fig. 1).

**Fig. 1:**
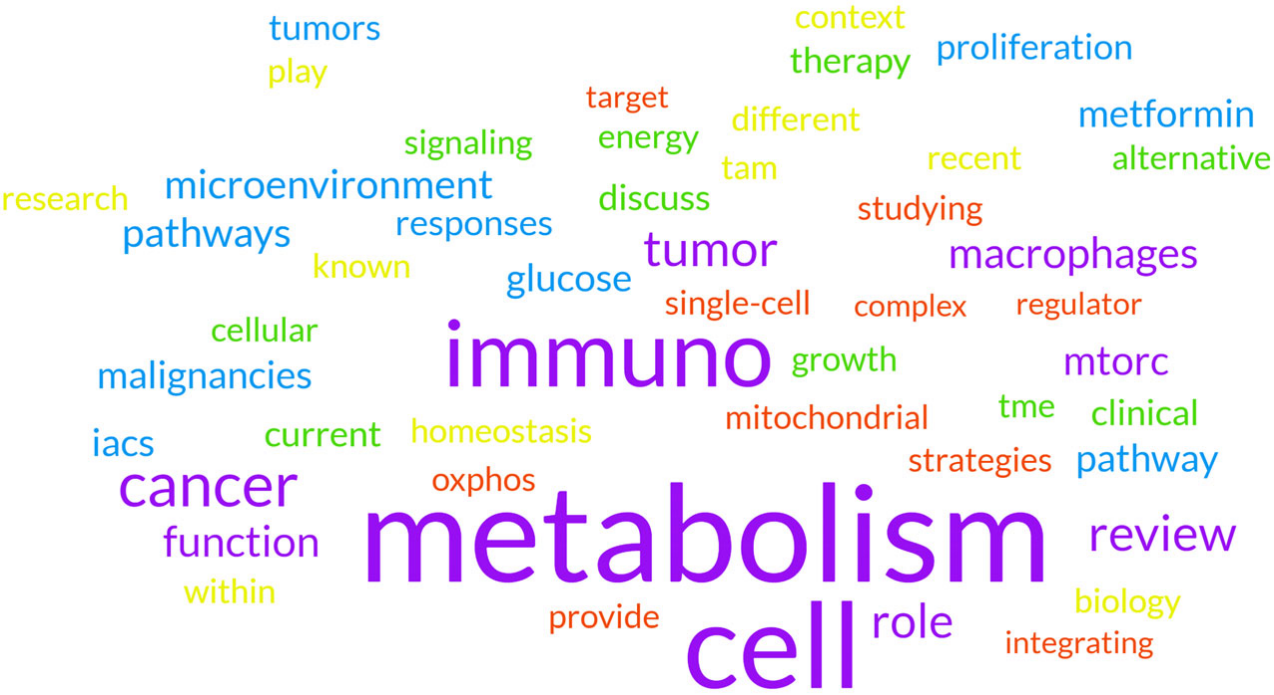
Word cloud generated from words used in the abstracts for this issue.

Deciphering the complex code of immune metabolism in cancer can hold the key to unleashing the full potential of immunotherapies. Studying immunometabolism within the tumor microenvironment is crucial for tailoring effective cancer therapies. This microenvironment hosts dynamic interactions between cancer and immune cells, shaping the success of immune responses. By unraveling these metabolic intricacies, we can identify personalized therapeutic targets, predict responsiveness to immunotherapies, and develop precision medicine approaches. In essence, studying immunometabolism in the proper tumor microenvironment is a strategic imperative for advancing cancer research and treatment.

The notion of understanding the molecular dialogues that shape the immune responses within tumors is put forward by Berkers et al. who highlight challenges and novel methods to study metabolism taking into account the importance of the microenvironment.

Metabolic inhibitors, a promising class of anticancer agents, disrupt crucial cellular processes to hinder cancer cell growth and survival. By selectively targeting specific metabolic pathways, these inhibitors induce metabolic stress, impacting cancer cells while sparing normal ones. This precision strategy holds significant potential for advancing innovative and more effective cancer treatments. A focal point in clinical trials has been the disruption of mitochondrial Complex I, identified as a potent strategy against cancer. The review from Bost et al. comprehensively outlines both the rationale and potential pitfalls associated with such approaches. Recognizing that impeding mitochondrial metabolism may affect not only cancer cells but also immune and nonmalignant cells, the review aims to provide a nuanced understanding of the challenges and opportunities in this evolving therapeutic landscape.

Recent research has spotlighted B lymphocytes, traditionally known for their role in humoral immunity, as exerting either pro‐ or antitumor activities [6]. Their unique ability to modulate the tumor microenvironment and orchestrate targeted immune responses marks a paradigm shift in our understanding of cancer surveillance. Two reviews in this special issue focus on the metabolism of B cells on their function. Kabanova et al. highlight the role of glucose metabolism, while the review by Peeters et al. focuses on the importance of lipid metabolism in B cell biology and function.

Chimeric antigen receptor T‐cell (CAR‐T) therapy is a revolutionary form of immunotherapy where a patient's T cells are genetically modified to express a receptor (CAR) that enables them to specifically target and destroy cancer cells. Their remarkable success in treating certain hematological cancers highlights their potential to revolutionize cancer treatment, offering a personalized and highly effective strategy. CAR‐T cells stand at the forefront of immunotherapy, holding promise for extending their impact to a broader spectrum of cancers and transforming the landscape of oncological care. It is now also realized that the frequent failure of CAR‐T treatment [7, 8] may be intrinsically linked with suboptimal differentiation and metabolic rewiring, and recent reports suggest ways to address this [9, 10]. The review by Taylor et al. underlines the importance of metabolism in the generation, production, and function of those T cells.

Tumor‐associated macrophages (TAMs) emerge as pivotal players in cancer control. These immune cells within the tumor microenvironment influence various aspects of cancer progression, including promoting or inhibiting tumor growth, function of other immune cells, angiogenesis, and metastasis. TAMs not only serve as potential biomarkers for prognosis but also represent targets for modulating the immune landscape, offering a strategic avenue in the pursuit of effective cancer treatments. The review of Mazzone et al. describes the metabolic features of the different TAM populations and their role in cancer development and treatment.

From a wider perspective, the enormous excitement in the overlapping fields of immune‐ and cancer metabolism stems from the expectation that once we understand the underlying mechanics, we may be able to fully harness the promises of cellular cancer immunotherapy. Given the current rate of discoveries and new strategies that involve the metabolism of both cancer and immune cells, we believe there is reason for optimism. We hope the articles in this thematic issue will inform the readers on several state‐of‐the‐art developments and emphasize the necessity of considering metabolism in the specific microenvironment. Assembling the collection has been a gratifying experience. Our heartfelt appreciation goes to our colleagues who promptly and willingly contributed to both writing and reviewing.

## Conflict of interest

The authors declare no conflict of interest.

## Author contributions

EE and J‐ER wrote the manuscript.
